# Development and psychometrics of the English version of the Itch Cognitions Questionnaire

**DOI:** 10.1371/journal.pone.0230585

**Published:** 2020-03-19

**Authors:** Carolyn J. Heckman, Christina Schut, Mary Riley, Anke Ehlers, Rodrigo Valdes-Rodriguez, Jörg Kupfer, Uwe Gieler, Jerod L. Stapleton

**Affiliations:** 1 Cancer Prevention and Control, Rutgers Cancer Institute of New Jersey, New Brunswick, NJ, United States of America; 2 Institute of Medical Psychology, Justus-Liebig-University Gießen, Gießen, Germany; 3 Department of Experimental Psychology, University of Oxford, Oxford, United Kingdom; 4 Department of Dermatology, University of Virginia, Charlottesville, VA, United States of America; 5 Department of Dermatology, Justus-Liebig-University Gießen, Gießen, Germany; 6 Department of Health Behavior, University of Kentucky, Lexington, KY, United States of America; University of Twente, NETHERLANDS

## Abstract

**Background:**

The aim of this study was to validate the English version of the Itch Cognition Questionnaire in a sample of patients with chronic itch due to psoriasis or atopic dermatitis. An English-language version of an instrument assessing itch-related cognitions is needed since cognitions can contribute to a worsening of itch, and chronic itch is prevalent in English-speaking counties and internationally.

**Methods:**

The German Itch Cognitions Questionnaire was translated into English, and cognitive interviewing was conducted to finalize item wording. Internal and test-retest reliability, item discrimination, responsiveness to change, and construct, convergent, and discriminant validity were assessed in a national sample of 137 individuals with chronic itch due to atopic dermatitis or psoriasis recruited online.

**Results:**

Internal reliability was high with Cronbach’s alphas of 0.93 for the Catastrophizing subscale and 0.88–0.90 for Coping. The Pearson’s correlation assessing 1-month test-retest reliability for the Catastrophizing subscale was *r* = 0.62 and for the Coping subscale was *r* = 0.61. The corrected item-total correlation revealed that items were relatively consistent with the scores for the subscales (with correlations ranging from 0.58 to 0.79), indicating very good item discrimination. Results of factor analysis, convergent and discriminant, and responsiveness to change analyses provided evidence for validity.

**Conclusions:**

This study showed good psychometric characteristics of the English version of the Itch Cognitions Questionnaire. We suggest that future studies investigate the use of the measure in clinical practice to assist with treatment planning and outcome assessment related to itch as well as address study limitations such as sampling and replication.

## Introduction

Chronic pruritus, which refers to itch lasting six weeks or longer, is common, particularly among individuals with certain common skin conditions such as atopic dermatitis (eczema) or psoriasis. In many studies, it has been shown that itch and scratching are related to psychological variables such as stress, personality traits, or emotions [1–4]. Also, cognitive reactions to external demands such as stress are thought to play a role in the worsening or improvement of chronic itch [5]. Indeed, the role of cognitive factors like attention or expectancies for perceived itch have been studied in recent years [6–9]. Catastrophic cognitions about itch (e.g., “The itching will never stop”) have been experimentally shown to increase severity of itch [10]. Itch-related coping has been found to be related to itch intensity in patients with atopic dermatitis [2]. Findings such as this make it reasonable to also measure itch cognitions on a regular basis in dermatology practice in order to offer and provide the best possible treatment to patients with chronic itch. Unfortunately, available questionnaires seem to address generic skin conditions rather than cognitive and behavioral coping with itch specifically and are not available in English.

The Itch Cognition Questionnaire (ICQ) [11] offers the possibility to efficiently measure coping with itch and itch catastrophizing. The questionnaire was developed in Germany with two clinical samples of patients with atopic dermatitis. It builds on cognitive theories suggesting that psychological distress and corresponding body sensations are enhanced by catastrophizising and unhelpful coping [12,13]. For example, in pain patients, a measure of coping and catastrophizing cognitions predicted subjective pain severity and distress [14]. The ICQ showed good to excellent reliability, factorial validity, and correlations with measures of distress and scratching. The questionnaire was sensitive to the effects of treatment [15]. It has been translated to Japanese and Dutch and validated in these languages already [8,16]. However, thus far, the questionnaire has not been available in English. The aim of the current study was to develop and validate an English-language version of the existing ICQ. It was hypothesized that the English version of the measure would demonstrate adequate reliability and validity according to standard psychometric guidelines. Since the questionnaire was designed to be sensitive to changes with treatment [11], a moderate test-retest reliability was expected.

## Materials and methods

### Study phases

This study was approved by the Fox Chase Cancer Center Institutional Review Board, protocol #16–9006. Digital or written consent was provided. The first author of the ICQ (AE) and a colleague translated the Itch Cognitions Questionnaire from German into English. Two authors (CS and JK) back-translated it into German. Then two authors reviewed the back translation and made changes accordingly (AE and UG).

After translation was complete, individual cognitive interviews were conducted with 10 individuals with psoriasis and 7 individuals with AD to gather their feedback about the English version of the ICQ in terms of item wording, understandability, clarity, and relevance. First, participants were asked to complete the measure as they normally would. It took cognitive interview participants an average of 2.23 minutes to complete the 20-item ICQ. Then the items were re-read one at a time, and participants were queried as to any feedback for each individual item. Participants commented on what was potentially problematic about some items and provided suggestions for improvement. Sessions were digitally recorded and transcribed, and a moderator took notes. Participants for interviews were a convenience sample recruited from patients from a university itch center, the local community, staff at a community hospital and a cancer center, and via word of mouth in the Philadelphia, Pennsylvania area.

Based on feedback from interviews, several items were refined: “it” was changed to “the itching” for several items; “look disfigured” was changed to “look horrible”; “cannot bear it” was changed to “cannot stand it”; “I should think about something cool” was changed to “I should try imagining that my skin feels cool”; and “to avoid scratching” was added to “I should keep my hands busy”.

Once the items were finalized, psychometric testing was conducted. Participants in the psychometric phase were primarily recruited from the panel of a market research company, Marketing Systems Group, and also took part in an intervention study (see below). Panelists who reported that they had AD, eczema, or psoriasis were presented with generic links to several potential surveys including the current one (“Tell us your opinion”). Panelists who clicked on the study link were then asked to complete a brief screener survey to determine eligibility. Eligibility criteria for both the interview and psychometric phases of the study included: adults 18 and older with a self-reported diagnosis of psoriasis or AD, itch for at least six weeks, fluent in English, living in the USA, internet access, and able to provide informed consent. Eligible and consented individuals with psoriasis or AD completed the full survey online. Once the survey was completed, patients were given access to an educational website designed to improve itch-related quality of life (QoL), results of which will be reported in a separate paper. After four weeks, participants were invited to complete a follow-up survey. All participants in the interview and psychometric phases received electronic gift-cards for their participation.

### Measures

All measures other than demographics were scored with higher values denoting more severe symptoms or impairment except for the Itch Cognitions Coping subscale, the Morisky Medication Adherence Scale, and Perceived Efficacy in Patient-Physician Interactions.

#### Demographics

Standard demographic characteristics assessed were sex, age/birthdate, race/ethnicity, education, income, employment, and insurance status.

#### Itch cognitions

The 20-item Itching Cognitions Questionnaire [11], validated initially in German, includes reliable (Cronbach’s alphas [*a*s] = 0.78 to 0.93) and valid problem-focused coping and catastrophizing (helpless coping) subscales [8,11,17,18]. It comprises 20 items, which are answered on a scale from 0 to 4 (“The thought never occurs”, “The thought always occurs”). Factor analyses of the German version in two samples of patients with atopic dermatitis supported the two-factor structure [11]. The catastrophizing subscale correlated with measures of illness-related and itch-related distress and the frequency and intensity of scratching; the coping scale correlated negatively with the duration of the illness and frequency of scratching, and positively with itch-rated distress. These effects remained significant when severity of skin manifestations was controlled. The scales were sensitive in measuring changes with therapy and differences between different therapeutic approaches [11,15].

#### For assessing convergent validity

*5-D pruritus scale*. The 5-D Pruritus Scale was used to assess duration, degree, direction, disability, and distribution of itch. Scores range from 5 (no pruritus) to 25 (severe pruritus). The 5-D Pruritus Scale is a reliable, multidimensional measure of itching that has been validated in patients with chronic pruritus to able to detect changes over time [19].

*Itch-related quality of life*. The 22 item ItchyQoL questionnaire focuses on symptoms, functional limitations, and emotions related to itch. Higher scores indicate more impairment to QoL due to itch. The ItchyQoL has demonstrated internal consistency, reproducibility (intra-class correlation coefficients = 0.84–0.91), discriminant validity, and responsiveness to change in several populations [20].

*Itch severity scale*. A numerical rating scale was used to assess itch severity (0–10) on a weekly basis.

*Scratch intensity and impact scale*. This 15-item measure was developed for the study. Items such as “I scratch a lot” and “My scratching is under control” were rated from never to always for the last seven days. Cronbach’s alphas in this sample were 0.93 at baseline and 0.92 at 1-month follow up.

*Sleep-related itch and scratch*. This 30-item measure was developed for the study. Items such as “Feeling tired makes me scratch more” were rated from never to always for the last seven days. Cronbach’s alphas in this sample were 0.98 at baseline and at 1-month follow up.

*Perceived stress*. Since stress can be an itch trigger, itch can cause stress, and the online intervention included stress-management training, the validated 4-item Perceived Stress Scale was included (*a*s = 0.60–0.82) [21]. Cronbach’s alphas in this sample were 0.63 at baseline and 0.67 at 1-month follow up.

#### For assessing discriminant validity

*Morisky Medication Adherence Scale (8-Item) MMAS-8-itch specific*. Treatment adherence is essential for successful management of chronic itch and the conditions that cause it. The Morisky Medication Adherence Scales (MMAS-8) is a validated scale that estimates the risk of medication non-adherence and consists of eight items assessing reasons for non-adherence. These items have been adapted for itch. The MMAS-8-IS score ranges between 0 (highly non-adherent) and 8 (highly adherent) [22,23].

*Perceived Efficacy in Patient-Physician Interactions (PEPPI-5)*. Successful patient-provider interactions facilitate appropriate medical treatment of acute and chronic conditions. The PEPPI-5 measures self-efficacy of patients to interact with their providers (a = 0.92) [24]. Cronbach’s alphas in this sample were 0.90 at baseline and 0.90 at 1-month follow up.

### Statistical analyses

The statistical analyses were carried out using IBM SPSS Statistics Version 25. The psychometric characteristics listed below were assessed. Baseline data (i.e., data obtained before participation in the online intervention) were used unless otherwise specified.

#### Reliability

Internal reliability, the degree of consistency of the item responses across individual subjects taking into account random error from item selection, was evaluated using Cronbach’s alpha coefficient for the subscales [25]. Coefficient scores >0.70 generally indicate good internal reliability [26]. Test-retest reliability (stability) of the questionnaire was measured Pearson’s correlation of the scores on the first and second (one month later) survey completions [25]. A portion of the sample that either did not visit the intervention website or only visited the homepage/contact us page were used in this analysis only (n = 21).

#### Item discrimination

Item discrimination was assessed utilizing corrected item-total correlations. Individual item scores were correlated to the subscale score without that item. If there had been low correlations, the item would be considered for removal.

#### Construct validity (domain structure)

We tested the dimensional structure of the final scales and items within the scale using exploratory factor analysis (principal component analysis with a varimax rotation) [27].

#### Convergent validity

Convergent validity was assessed using correlations between the ICQ and other measures anticipated to be associated with itch-related cognitions including the Itch Quality of Life Scale, 5D Pruritus Scale, a numeric rating scale of itch severity, the Sleep Related Itch and Scratch Scale, Scratch Intensity and Impact Scale, and Perceived Stress Scale.

#### Discriminant validity

Correlations between the ICQ and the presumably unrelated measures (MMAS, PEPPI) were calculated. Correlations with discriminant scales were expected to be lower than for convergent scales.

#### Responsiveness to change

A two-sided, paired samples t-test to evaluate pre- to post-intervention changes on the ICQ as a result of the online educational website was performed [25].

### Ethics

The study was conducted in concordance with the Declaration of Helsinki. Institutional Review Board (IRB) approval was received from the IRB of Fox Chase Cancer Center/Temple University. Participants provided informed consent.

## Results

### Characteristics of the sample

From the 248 individuals who completed the initial screening questionnaire, 173 (69.76%) were eligible based on the aforementioned criteria, 164 (94.80%) consented to participate, 137 (83.54%) completed the baseline survey, and 108 (78.83%) completed the one-month follow-up survey. Baseline participants included 69 individuals with AD and 68 with psoriasis. Demographic characteristics are provided in Table 1. Since the psoriasis and AD patients did not differ significantly on any of the demographic or other study variables at baseline or follow up, the samples were combined for subsequent analyses.

**Table 1:** Sample demographic characteristics.

	Interview Sample % (n of 17)	Psychometric Sample		
		Psoriasis % (n of 68)	Eczema % (n of 69)	Total % (n of 137)
**Disease**				
Psoriasis	59 (10)	100 (68)		49.6 (68)
Eczema	41 (7)		100 (69)	50.4 (69)
**Sex**				
Male	35 (6)	42.6 (29)	44.9 (31)	43.8 (60)
Female	65 (11)	57.4 (39)	55.1 (38)	56.2 (77)
**Age**				
18 to 35	53 (9)	41.2 (28)	33.3 (23)	37.2 (51)
36 to 55	30 (5)	44.1 (30)	50.7 (35)	47.4 (65)
56 and older	18 (3)	14.7 (10)	15.9 (11)	15.3 (21)
**Race/Ethnicity**				
White	53 (9)	86.8 (59)	75.4 (52)	81.0 (111)
Non-white/Hispanic	48 (8)	13.2 (9)	24.6 (17)	19.0 (26)
**Education**				
High school/GED	18 (3)	20.6 (14)	21.7 (15)	21.2 (29)
College or some college	59 (10)	66.2 (45)	62.3 (43)	64.2 (88)
Masters, professional, or doctorate	24 (4)	13.2 (9)	15.9 (11)	14.6 (20)
**Employment**				
Full time	65 (11)	67.6 (46)	56.5 (39)	62.0 (85)
Other than full time	35 (6)	32.4 (22)	43.5 (30)	38.0 (52)
**Insurance**				
Medicaid	12 (2)	17.6 (12)	14.5 (10)	16.1 (22)
Medicare	12 (2)	14.7 (10)	8.7 (6)	11.7 (16)
Private	77 (13)	61.8 (42)	66.7 (46)	64.2 (88)
No insurance	0 (0)	5.9 (4)	10.1 (7)	8.0 (11)
**Marital Status**				
Never Married	65 (11)	22.1 (15)	36.2 (25)	29.2 (40)
Married/Cohabitating	24 (4)	55.9 (38)	49.3 (34)	52.6 (72)
Divorced/ Widowed/Separated	12 (2)	22.1 (15)	14.5 (10)	18.2 (25)
**Income**				
Less than $50,000	24 (4)	35.3 (24)	39.1 (27)	37.2 (51)
$50,000 - $99,999	29 (5)	45.6 (31)	42.0 (29)	43.8 (60)
$100,000 or more	24 (4)	16.2 (11)	15.9 (11)	16.1 (22)
Prefer not to answer	24 (4)	2.9 (2)	2.9 (2)	2.9 (4)
**Provider**				
Dermatologist	82 (14)	80.9 (55)	71.0 (49)	75.9 (104)
Other than Dermatologist	18 (3)	19.1 (13)	29.0 (20)	24.1 (33)

### Reliability

The Catastrophizing subscale showed high internal reliability of α = 0.93 at baseline and α = 0.93 at follow up. The Coping subscale’s reliability was α = 0.90 at baseline and α = 0.88 at follow up. The Pearson’s correlation assessing test-retest reliability for the Catastrophizing subscale was *r* = 0.62 (n = 21), and for the Coping subscale was *r* = 0.61 (n = 21).

### Item discrimination

The corrected item-total correlation revealed that items were relatively consistent with the overall score (with correlations ranging from 0.473 to 0.820) for the subscales indicating very good item discrimination. The corrected item-subscale correlations at baseline ranged from 0.653 to 0.794 for the Catastrophizing subscale and 0.575 to 0.746 for the Coping subscale and from 0.570 to 0.820 for the Catastrophizing subscale and 0.473 to 0.727 for the Coping subscale at follow up. In general, the Catastrophizing subscale showed better item discrimination than the Coping subscale.

### Construct validity

An exploratory factor analysis was conducted to determine factor structure. Two factors were identified based on eigenvalues above one (9.728 for Catastrophizing and 1.973 for Coping) and scree plot curve interpretation. Additional factors had low eigenvalues. The amount of variance explained was 48.64% for Catastrophizing and 9.87% for Coping. Table 2 provides the mean and standard deviations and factor loading of each item.

**Table 2:** Itch Cognitions Questionnaire (English version) psychometrics.

Final Item Wording (Subscale)	M(SD)		Rotated Factor 1 Loading—CAT	Rotated Factor 2 Loadings—COP
	Baseline (n = 137)	Follow up (n = 107)		
1. The itching will never stop. (CAT)	2.75 (0.91)	2.39 (0.99)	.705	
2. There is nothing I can do about the itching. (CAT)	2.44 (0.98)	2.18 (0.95)	.810	
3. I will scratch myself again until I look horrible. (CAT)	2.24 (1.16)	2.15 (1.08)	.768	
4. I should try to relax. (COP)	2.65 (0.98)	2.67 (0.92)	.308	.629
5. The itching will get worse and worse. (CAT)	2.54 (1.02)	2.21 (1.02)	.705	.348
6. I must distract myself from the itching. (COP)	2.80 (1.00)	2.49 (0.98)	.454	.548
7. I cannot stand the itching. (CAT)	2.98 (0.99)	2.61 (1.04)	.775	
8. I will definitely not have a moment’s peace again today/tonight. (CAT)	2.16 (1.15)	1.95 (1.09)	.707	.429
9. I must pay better attention to what triggers the itching. (COP)	2.53 (1.06)	2.58 (0.92)		.666
10. I should try imaging that my skin feels cool. (COP)	2.06 (1.24)	2.03 (1.10)		.818
11. I will resist the itching and will not scratch myself. (COP)	2.36 (1.03)	2.17 (0.94)		.710
12. There have been times when the itch was much worse. (COP)	2.63 (0.96)	2.57 (0.97)		.636
13. I must suppress the itching. (COP)	2.64 (1.02)	2.54 (0.92)	.312	.634
14. All the set-backs with my itch make me desperate. (CAT)	2.13 (1.19)	2.05 (1.16)	.639	.498
15. The itching is going to drive me mad. (CAT)	2.45(1.19)	2.30 (1.10)	.795	
16. I will scratch myself again until I bleed. (CAT)	2.24 (1.27)	2.21 (1.11)	.695	
17. I should talk with someone to distract myself from the itching. (COP)	2.17 (1.18)	2.10 (1.08)	.392	.696
18. My skin will definitely look awful tomorrow. (CAT)	2.46 (1.12)	2.23 (1.16)	.708	.350
19. I should keep my hand busy to avoid scratching. (COP)	2.42 (1.17)	2.42 (1.00)	.502	.639
20. I could put something cool on my skin. (COP)	2.58 (1.17)	2.51 (1.00)	.333	.602

CAT = catastrophizing subscale; COP = coping subscale.

### Convergent and discriminant validity

The Catastrophizing and Coping scales showed good convergent validity by correlating significantly with other relevant itch and coping measures such as the Itch Quality of Life scale, 5-D Pruritus scale, Itch Severity, Sleep Related Itch and Scratch, Scratch Intensity and Impact Scale, and Perceived Stress scale (all *p*s < 0.01) (Table 3). The inter-correlations of the Catastrophizing and Coping subscales were r = 0.68 in the current sample. In terms of discriminant validity, the Coping subscale did not correlate significantly with the Perceived Efficacy in Patient Physician Interactions scale, but the Catastrophizing subscale did (p < 0.01). As expected, the scales did not correlate with the MMAS.

**Table 3:** Correlations between itch cognition subscales and other scales at baseline.

Other Scales	Possible Range	Mean (SD)	Catastrophizing	Coping
**Convergent**				
Coping	0–40	24.82 (7.81)	0.69**	
Catastrophizing	0–40	24.39 (8.66)		0.69**
Itch Quality of Life	22–110	79.70 (15.70)	0.82**	0.63**
5D Pruritus Scale	5–25	15.46 (2.87)	0.58**	0.42**
Perceived Stress Scales	0–16	7.40 (3.10)	0.38**	0.20**
Itch Severity	1–10	6.79 (1.71)	0.59**	0.46**
Scratch Intensity and Impact Scale	0–52	32.08 (9.56)	0.77**	0.59**
Sleep Related Itch and Scratch	0–64	37.50 (17.11)	0.73**	0.67**
**Discriminant**				
Morisky Medication Adherence Scale	0–8	4.75 (2.01)	-.160	0.06
Perceived Efficacy in Patient–Physician Interactions Scale	5–25	19.15 (4.24)	0.02	0.27**

** *p* < 0.01 (by Pearson’s *r*).

### Responsiveness to change

Regarding responsiveness to change, there was a decrease in scores after exposure to the educational website on the Catastrophizing subscale from baseline (M = 24.61, SD = 8.31) to follow up (M = 22.28, SD = 8.44). This difference of 2.33 points was significant *t*(106) = 3.339, *p* = .001. There was no significant difference between scores on the Coping subscale between baseline (M = 24.75, SD = 7.45) and follow up (M = 24.08, SD = 6.87).

## Discussion

The aim of this study was to develop and validate the English version of the Itch Cognition Questionnaire in a sample of patients with chronic itch due to psoriasis or atopic dermatitis. This study showed good psychometric characteristics of the English version of the Itch Cognitions Questionnaire. The two-factor-structure of the questionnaire is consistent with the German, Dutch, and Japanese versions. It is notable that the correlation between the two factors in the English version (*r* = 0.68) was much higher than in the German version (*r* = 0.26). However, we believe it is appropriate and informative to retain both factors.

Consensus-based guidelines on how to treat chronic itch [28] recommend assessing the impact of psychological factors on the worsening or maintenance of chronic pruritus. Thus, the Itch Cognition Questionnaire could be used on a regular basis in clinical practice in order to get a better insight into whether and which kind of psychological intervention should be offered to the patient. As outlined in previous studies [29,30], there are different psychological approaches that could be especially useful depending on whether irrational itch-related beliefs, compulsive scratching behavior, itch-related stress, or all of them are in the focus [30].

Strengths of the study include the mixed methods and the use of a national sample for the psychometric analyses. We suggest that future research address study limitations by comparing administration modalities (online vs. paper) and recruiting a larger sample of more heterogeneous participants with objectively-verified diagnoses from other English-speaking populations, particularly for replication of factor and test-retest analyses and associations with other measures such as severity and duration of disease, other comorbidities, and treatments. Additional cognitive interviews with participants from other parts of the country with different demographic characteristics (e.g., older, lower socioeconomic status) from the current interview sample could also be useful.
